# [Expression of Concern] miR-30a inhibits glioma progression and stem cell-like properties by repression of Wnt5a

**DOI:** 10.3892/or.2025.8983

**Published:** 2025-09-04

**Authors:** Yonghong Zhang, Zhi Wu, Lichao Li, Min Xie

Oncol Rep 38: 1156–1162, 2017; DOI: 10.3892/or.2017.5728

Following the publication of this paper, it was drawn to the Editor's attention by a concerned reader that the control GADPH western blots shown in Fig. 5D were strikingly simillar to three lanes in the GAPDH panel shown in Fig. 4D, even though the experimental conditions reported in these figure parts were different, suggesting that one of these figures may have been assembled incorrectly.

The authors were contacted by the Editorial Office to offer an explanation for this apparent anomaly in the presentation of the data in this paper; however, up to this time, no response from them has been forthcoming. Owing to the fact that the Editorial Office has been made aware of potential issues surrounding the scientific integrity of this paper, we are issuing an Expression of Concern to notify readers of this potential problem while the Editorial Office continues to investigate this matter further.

